# Assessing decarbonization strategies and industrial symbiosis in the chemical and waste-to-energy sector

**DOI:** 10.1111/jiec.13616

**Published:** 2025-01-23

**Authors:** Maria Schnyder, Jing Huo, Stefanie Hellweg

**Affiliations:** 1 Chair of Ecological Systems Design, Institute of Environmental Engineering, ETH Zürich, Zürich, Switzerland; 2 National Centre of Competence in Research (NCCR) Catalysis, ETH Zürich, Zürich, Switzerland

**Keywords:** carbon capture and storage (CCS), carbon capture and utilization (CCU), carbon footprint analysis, ethylene production, industrial ecology, waste-to-energy

## Abstract

Swiss waste-to-energy (WtE) plants are required to capture their CO_2_ emissions by 2050 to meet the net-zero climate target, with options for underground storage (carbon capture and storage [CCS]) or utilization (carbon capture and utilization [CCU]). This opens up a synergistic opportunity for the petrochemical industry to utilize the captured CO_2_ as a feedstock, potentially helping both sectors reduce their carbon footprints. We conducted a prospective carbon footprint analysis on various net-zero strategies within the Swiss WtE plants (CCU and CCS) and German ethylene production (CO_2_-based ethylene, bio-ethylene, and fossil ethylene with CCS), including scenarios of industrial symbiosis. While focusing on these two countries, the findings offer valuable insights applicable to similar sectors in other regions. All assessed pathways reduce the carbon footprint by at least 60% relative to the reference scenario (no carbon capture in WtE plants and fossil ethylene production). Bio-ethylene and direct air capture–based ethylene combined with CCS in WtE exhibit the lowest climate change impacts, achieving net negative emissions when powered by renewable electricity. However, these pathways all come with trade-offs: The availability of sustainable biomass and low-carbon electricity is limited, and future resource competition may delimit the penetration of these technology combinations. CCS in ethylene production plants could reduce emissions while utilizing existing infrastructure but does not eliminate emissions from fossil fuel extraction. Ethylene produced with CO_2_ from WtE plants could be a viable interim solution until CCS barriers are overcome.

**Supplementary Information:**

The online version of this article (doi:10.1111/jiec.13616) contains supplementary material, which is available to authorized users.

## INTRODUCTION

Under the Paris Agreement, nations committed to limiting global warming to well below 2°C above pre-industrial levels (UNFCCC, 2015). To achieve this goal, countries, including Switzerland, have pledged to drastically reduce greenhouse gas (GHG) emissions and reach carbon neutrality by 2050 (Bundesrat, 2021; EU, 2020). All industries need to reduce their emissions by 80%–95%, which poses challenges in hard-to-abate sectors because reducing emissions is costly and the technological solutions are not commercialized (EC, 2011; IEA, 2020). By capturing CO_2_ from flue gas and storing it underground, emissions from these industries can be further reduced (Galán-Martín et al., 2021). Waste-to-energy (WtE) and chemical industries are two examples of hard-to-abate sectors. WtE plants in Switzerland incinerate around 4 million tonnes of municipal solid waste per year, producing approximately 3% of the electricity and 36% of district heat of the country (BFE, 2023; InfraWatt et al., 2023; VSBA, 2023). At the same time, they emit around 2.1 million tonnes of fossil GHG emissions per year, about a third of the industrial GHG emissions in Switzerland (BAFU, 2022b, 2023; VBSA et al., 2021). Wiprächtiger et al. (2023) identified carbon capture and storage (CCS) as the most reliable and efficient strategy for decarbonizing the WtE sector since alternative decarbonization methods are limited. The amount of Swiss residual waste in 2050 is projected to stay within the range of 96%–116% of the waste volume of 2015 (Prognos, 2018). To reach the net-zero target, the Swiss Association of Waste Treatment Plants has committed to capturing the CO_2_ emissions from all WtE plants that emit more than 100,000 tonnes of CO_2_ annually by 2050. A pilot carbon capture plant is planned to operate by 2030 (UVEK & VBSA, 2022). CCS in WtE plants has the potential to generate net negative emissions because half of the captured CO_2_ is biogenic (Pour et al., 2018). These negative emissions, together with other negative emission technologies), are essential to limit global warming to 1.5°C (IEA, 2021).

Alternatively, captured CO_2_ can be utilized to produce chemicals, synthetic fuels, or building aggregates (carbon capture and utilization [CCU]) (IEA, 2023a). Globally, the chemical industry is the primary industrial fossil fuel consumer and the third-largest industrial GHG contributor (IEA, 2018). To reach the net-zero target, low-carbon production pathways must be implemented, including utilizing renewable energy and alternative carbon feedstocks, such as biomass or captured CO_2_ (Chen & Patel, 2012; Huo et al., 2023; Keller et al., 2020). Since Switzerland does not have a large petrochemical industry, the chemical industry in the neighboring country, Germany, is considered a potential consumer of the captured CO_2_ from Swiss WtE plants. The German chemical industry contributes to approximately 23% of industrial GHG emissions (Destatis, 2018; Gehrke et al., 2018; VCI, 2019). In Germany, ethylene production reached 4.3 million tonnes in 2022 and was associated with 10% of the GHG emissions within the chemical industry (Achtelik et al., 2020; Fleiter et al., 2013; VCI, 2023).

Life cycle assessment (LCA) is an effective tool for evaluating environmental impacts and trade-offs from a systems perspective. It provides insights that support environmentally informed policy-making and decision-making processes (Hellweg & Milà i Canals, 2014). Past LCA studies in the CCU/CCS domain primarily concentrated on the effect of reducing carbon emissions in a specific product or industry. On the CCS side, Tang and You (2018) and Bisinella et al. (2022) evaluated the environmental effects of CCS in two WtE plants located in China and Denmark. Meanwhile, Poretti and Stengler (2022) and Pour et al. (2018) calculated the European and global potential of CCS in WtE plants and estimated the resulting amount of negative emissions. On the CCU side, several studies have investigated the environmental impacts of methanol and olefins produced with renewable feedstock compared to fossil feedstock (Kajaste et al., 2018; Kätelhön et al., 2019; Keller et al., 2020; Zhao et al., 2018). Christensen and Bisinella (2021) and Hoppe et al. (2018) studied the environmental impact of chemicals derived from CO_2_ captured in WtE plants. All of these studies conducted LCAs that focused specifically on the environmental impact per tonne of waste or tonne of chemicals. In contrast, Lausselet et al. (2017) compared four different waste treatment scenarios in Norway and included multiple functions of the system in their analysis. A similar approach is used in this study, with a focus on two industries within the framework of industrial symbiosis.

Industrial symbiosis can be defined as resource-sharing between different industries and includes the exchange of physical goods (materials, water, energy, by-products) with the goal of enhancing energy and resource efficiency while reducing waste and emissions output (Korhonen, 2001). For example, CO_2_ as a waste of industrial point sources can become a valuable resource for chemical production. Initially, industrial symbiosis was studied at the industrial park level, but now there are more studies extending to country and regional levels. (Duraccio et al., 2015; Golev et al., 2014; Nørstebø et al., 2012; Sokka et al., 2011).

The main objective of this study is to assess the carbon footprint of four decarbonization scenarios involving the WtE industry and ethylene production from a life cycle perspective. The German petrochemical industry could potentially become a consumer of carbon captured from Swiss WtE plants, which could provide insights into cross-border industrial symbiosis opportunities. Potential synergies and trade-offs can be identified by analyzing the combined effects of decarbonization strategies in both industries, not only in Germany and Switzerland but also in other locations with similar technologies. This comprehensive approach can offer valuable perspectives for policymakers and stakeholders to design effective policies and incentivize collaboration between industries.

## MATERIALS AND METHODS

This study used a prospective carbon footprint analysis to model the climate impact of different decarbonization strategies in WtE plants and ethylene production. The following section describes the goal and scope, presents the assessed technologies, and discusses the various combinations of carbon mitigation pathways. The scenarios were set within a net-zero scenario in 2050.

### Carbon footprint analysis

Net-zero strategies were developed and assessed for Swiss WtE plants and German ethylene production facilities. Swiss WtE plants need to capture CO_2_ from flue gases for underground storage or downstream utilization, while German ethylene production facilities need to adopt alternative low-carbon production pathways. Several scenarios were assessed to understand the potential carbon benefits and develop effective strategies for carbon reduction.

To ensure a standardized basis for comparison across different scenarios, the functional unit (FU) in this study was defined as:

- 132,876 tonnes of waste (52% biogenic carbon, LHV: 11.8 GJ/t)
- 35,160 tonnes of ethylene
- 59,406 MWh electricity
- 124,235 MWh district heat

The FU refers to the waste treated by an average Swiss WtE plant in a year and includes the electricity and heat produced during the incineration of the waste (Rytec, 2023). If the CO_2_ is captured and the WtE plant reduces its electricity output, the FU is maintained by adding electricity from the Swiss grid into the system to compensate for this energy penalty.

The amount of ethylene in the FU refers to the theoretical maximum volume of ethylene that can be produced through the methanol-to-olefin (MTO) process with the CO_2_ that is captured by an average Swiss WtE plant in 1 year. In scenarios where captured CO_2_ from WtE is stored underground instead, the same amount of ethylene is produced from other CO_2_ sources (direct air capture [DAC]W) or biomass feedstock to meet the FU requirements.

The total amount of CO_2_ captured was evaluated using the method provided in the official agreement between the WtE plant operators and the Federal Government (see Supporting Information S-1) (UVEK & VBSA, 2022; VBSA et al., 2021).

**FIGURE 1:**
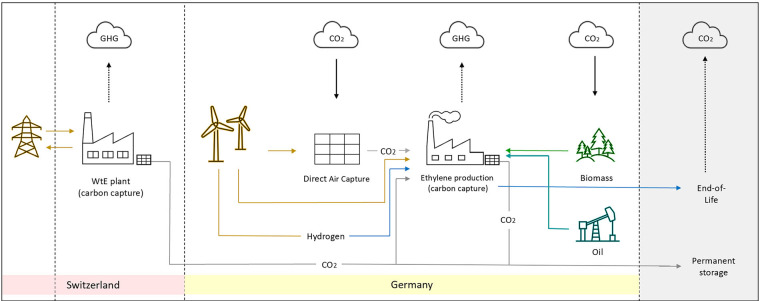
System boundaries with different decarbonization strategies: (a) Captured CO_2_ from WtE is either used in ethylene production or permanently stored. (b) The methanol-to-olefin (MTO) production process with e-methanol requires captured CO_2_, hydrogen, and electricity. (c) MTO production process with bio-methanol utilizes biomass and renewable electricity. (d) Carbon capture and storage (CCS) in steam cracking requires renewable electricity and steam. GHG, greenhouse gas.

The capture of CO_2_, ethylene production, the transportation of CO_2_ between the capture site and production or storage site, and end-of-life (EoL) incineration emissions of final products derived from ethylene (cradle-to-gate + EoL) were included in the system boundaries (Figure 1). This analysis does not account for any additional processing or use of the produced ethylene, as these are assumed to be the same across all scenarios. Furthermore, the construction of the facilities was excluded because the carbon emissions during the operational phase dominate those during construction in the assessed technologies (Reiter & Lindorfer, 2015; Tang & You, 2018; Terlouw et al., 2021).

Incineration was assumed as the EoL treatment method for ethylene-derived products. CO_2_ emissions from ethylene incineration were calculated stoichiometrically, assuming complete combustion (Equation 1). Per tonne of ethylene combusted, 3.14 tonnes of CO_2_ are emitted into the atmosphere.

1 $${{\mathrm{C}}_2}{{\mathrm{H}}_4} + 3 {{\mathrm{O}}_2} \to 2{\mathrm{C}}{{\mathrm{O}}_2} + 2 {{\mathrm{H}}_2}{\mathrm{O}}$$

The carbon footprint analysis was performed in *Brightway 2.0*, using the *ecoinvent 3.9* database with the cutoff allocation method (ecoinvent, 2023; Mutel, 2017; Wernet et al., 2016). Prospective LCA was conducted with *premise* 2.0.1, incorporating future technological and market shifts that are aligned with the Shared Socioeconomic Pathway 2 (SSP2) and Representative Concentration Pathway 1.9 (RCP1.9) scenarios from the IMAGE Integrated Assessment Model (Sacchi et al., 2022). The RCP1.9 represents the “net-zero” scenario that limits global warming to 1.5°C by 2100 while socioeconomic trends follow historical patterns (SSP2). A detailed description of the SSP2 scenario can be found in S-2.

Climate change impacts were assessed using the Global Warming Potential over 100 years (GWP100) with the *IPCC 2021* method (Forster et al., 2021). When fossil CO_2_ emissions were released into the atmosphere, they were projected to have a net climate change impact of +1 kg CO_2_-eq/kg fossil CO_2_. However, if they were stored permanently underground, they did not have a climate change impact (0 kg CO_2_-eq/kg fossil CO_2_). By contrast, biogenic and DAC-captured CO_2_ were reported as negative emissions with a value of −1 kg CO_2_-eq/kg CO_2_ when stored permanently. Biogenic CO_2_ emissions or CO_2_ emissions from DAC were assumed to have net-zero impacts if they were released into the atmosphere due to the initial CO_2_ uptake in biomass growth or sequestration in DAC. This is in line with the conventional assumption that CO_2_ emissions are classified as climate-neutral if the amount of CO_2_ emitted is balanced by the amount of CO_2_ sequestered from the atmosphere (IPCC, 2006). However, biogenic CO_2_ emissions may contribute to climate change because of the time lag between CO_2_ emission and resequestration (for details, see Section 2.5.3).

### Scenario overview

The reference scenario reflects current industry operations, without carbon capture in either the WtE industry or ethylene production. All other scenarios include carbon capture in WtE plants, and the carbon is either used in ethylene production (scenario 1) or permanently stored in Northern Europe (scenarios 2–4). Capturing carbon from WtE plants requires electricity and heat that can be sourced directly from the plant, which reduces its total electricity output (energy penalty). To compensate for this loss within the system, additional electricity from the Swiss grid is used. In scenarios 1 and 2, CO_2_-based methanol (e-methanol) is produced and converted into ethylene via the MTO process. In scenario 2, the CO_2_ for e-methanol production originates from the air (DAC) and not from the WtE plant, since CO_2_ emissions from WtE plants are captured and stored underground. Scenario 3 describes ethylene production with fossil feedstock, and process emissions during steam cracking are captured and stored underground. Bio-methanol, from the gasification of lignocellulosic biomass, is synthesized into bio-ethylene through the MTO process in scenario 4. The accounting of the EoL carbon emissions depends on the origin of the feedstock (see Section 2.1.). A summary is presented in Table Table 1. More details can be found in S-3, and S-5 describes two additional scenarios.

**TABLE 1:** Scenario overview.

Description	WtE: CO_2_ Capture	WtE: CO_2_ Storage	Ethylene: Feedstock	Ethylene: CCS
Reference	No	No	Naphtha	No
Scenario 1	Yes	No	CO_2_ (WtE) + H_2_	No
Scenario 2	Yes	Yes	CO_2_ (DAC) + H_2_	No
Scenario 3	Yes	Yes	Naphtha	Yes
Scenario 4	Yes	Yes	Biomass	No

Abbreviations: CCS, carbon capture and storage; WtE, waste-to-energy.

### Ethylene production pathways

#### Steam cracking

Steam cracking of hydrocarbons is the leading process for producing ethylene and is highly energy-intensive (Amghizar et al., 2017; Zimmermann & Walzl, 2009). The technical energy efficiency potential for steam cracking has already been maximized, and no further energy savings are expected by 2050 (Saygin et al., 2011). Naphtha is used as the primary feedstock for ethylene production in Germany, and ethylene production from steam cracking with naphtha was modeled after Keller et al. (2020). Life cycle inventories for methanol and ethylene production can be found in S-4.

#### MTO

The MTO process was selected as the main low-carbon chemical production pathway. Through a catalytic reaction at 495°C, methanol can be transformed into ethylene, making it a viable option for chemical production (Chang & Silvestri, 1977).

Methanol from coal or natural gas is typically used for commercial applications, but low-carbon methanol, such as e-methanol or bio-methanol, could serve as an alternative (Hurd et al., 2014; Zhao et al., 2018). E-methanol is derived from the direct hydrogenation of CO_2_ using green hydrogen generated through water electrolysis (IRENA, 2021).

Bio-methanol involves the gasification of biomass. Lignocellulosic biomass is the most abundant biomass worldwide (Zhang, 2008). For this study, cleft timber was considered the primary biogenic feedstock for bio-methanol production. Both the MTO process and bio-methanol require steam for production. Renewable steam production from biomass was considered in the context of the net-zero scenario and modeled based on Pérez-Uresti et al. (2019).

Chemical companies in Germany are investing in wind energy to replace electricity derived from fossil fuels in production processes, and several studies investigating chemical production from CCU have made the same assumption (BASF, 2023; Keller et al., 2020; Rosenthal et al., 2020). Therefore, electricity from offshore wind was used for chemical production in the net-zero 2050 scenario.

### Carbon capture

#### Carbon capture from waste-to-energy plants

The monoethanolamine (MEA) carbon capture method was chosen as the primary carbon capture technology in this study due to its prominence in literature and pilot projects (Poretti & Stengler, 2022). An overall carbon capture rate of 90% was assumed in this study (Li et al., 2016; Soltani et al., 2017; Tang & You, 2018; Zhao et al., 2013).

The heat and electricity for carbon capture can be sourced directly from WtE plants. This affects their net power output based on the specific plant and capture system configuration. Andersson (2020), Bisinella et al. (2021), Lausselet et al. (2017), and Magnanelli et al. (2021) each reported different levels of energy penalty. After consulting experts from the industry, a 50% reduction in electricity was assumed for this study (Bisinella et al., 2021). To compensate for these reductions, we assumed the electricity grid mix offsets the decrease in electricity production. The future Swiss grid electricity mix was modeled after Swiss Energy Perspectives’ *Zero* scenario, in which carbon neutrality is achieved (see Table 2; BFE, 2021).

**TABLE 2:** Electricity mix and carbon footprint overview.

Narrative	Description	Location	Carbon footprint (kg CO_2_-eq/MWh)	Electricity mix	Source for electricity mix
SSP2-RCP1.9 (main analysis)	Electricity production, wind, 1–3MW turbine, offshore	DE (Western Europe WEU)	9.434	100% Wind	premise IMAGE SSP2-RCP1.9 2050^a,b,c^
	Electricity CH high voltage—RCP1.9	CH	18.535	50% Hydro 38% Photovoltaic 5% Wind 2% WtE 2% Geothermal 1% Biogas 1% Natural gas 1% Solid biomass	Swiss Energy Perspectives 2050+, modeled in premise IMAGE SSP2-RCP1.9 2050^a,b,c,e^
SSP2-Base (Sensitivity)	Electricity CH high voltage—Base	CH	166.270	50% Hydro 15% Photovoltaic 3% Natural gas 2% WtE 1% Biogas 1% Geothermal 1% Wind 27% Imports	Swiss Energy Perspectives 2050+, modeled in premise IMAGE SSP2-Base 2050^a,b,d,e^
	Market for electricity, high-voltage	DE (WEU)	523.114	20% Natural gas 19% Hard coal 16% Lignite 15% Hydro 15% Wind 8% Photovoltaic 4% Nuclear 2% Geothermal 1% Solid biomass	premise IMAGE SSP2-Base 2050^a,b,d^

Abbreviations: CH, Switzerland; DE, Germany; RCP, Representative Concentration Pathway; SSP, Shared Socioeconomic Pathway; WtE, waste-to-energy.

^a^Sacchi et al. (2022).

^b^Stehfest et al. (2014).

^c^Rogelj et al. (2018)

^d^Riahi et al. (2017)

^e^BFE (2021).

The compensation of the heat used for carbon capture through heat pumps will probably be the strategy used by many Swiss WtE plants that already supply their surplus heat to district heat networks. However, there may be conditions where district heat networks are not favorable at the WtE site, and unused (burden-free) heat can be utilized for carbon capture. On the other hand, the additional demand for heat from WtE plants with carbon capture could inhibit the expansion of the district heat network, which would otherwise substitute conventional fossil heating. Thus, the impacts of carbon capture in WtE plants could be higher or lower, depending on the specific conditions.

#### Direct air capture

In this study, solid DAC was selected since it is the dominating technology in DAC projects and pilot plants (Viebahn et al., 2019). Atmospheric CO_2_ is more diluted than CO_2_ in flue gases of point sources, leading to substantially higher energy needs for capture (IEA, 2023b). Terlouw et al. (2021) provided data for DAC utilizing heat from by high-temperature heat pumps (HTHP), which was incorporated in this study. To avoid long transport distances, the DAC plant was assumed to be located close to the chemical production site.

#### Carbon capture in steam cracking

The carbon capture process in steam crackers for ethylene production was based on Hu et al. (2023) and Suviranta (2023), who analyzed post-combustion capture with MEA in ethylene production. No waste heat from the steam cracking process is available as energy for solvent recovery in carbon capture because the steam is fully utilized at the chemical plant (Suviranta, 2023). The additional steam in the SSP2-RCP1.9 scenario was presumed to be from bioenergy (wood).

The process emissions that can be captured during steam cracking were taken from Keller et al. (2020) and are 0.70 kg of CO_2_ per kg of ethylene. This value is similar to the 0.76 kg of CO_2_ per kg of ethylene from Suviranta (2023), who used industry data to model CCS in Finnish steam cracking plants.

#### CO_2_ transport and storage

Pipelines are the most viable method of transporting large volumes of CO_2_ over long distances (Becattini et al., 2022; Svensson et al., 2004). The electricity required for CO_2_ compression before and during pipeline transportation was obtained from Terlouw et al. (2021). Approximately 1.2% of the CO_2_ is lost per 1000 km of pipeline transport, and another 2% of CO_2_ is lost in storage due to leaking in the injection phase and off-site migration (Bisinella et al., 2022; IPCC, 2005). Most European carbon dioxide storage projects in the near future will be located in Northern Europe (Adomaitis & Kartit, 2023). For this study, a CO_2_ transport distance of 2000 km from Switzerland to a storage site in Northern Europe was assumed. Long-distance transport might be avoidable in the future since Switzerland is exploring its prospects for its own geological carbon dioxide storage site, and ongoing research aims to identify appropriate sites for permanent CO_2_ storage (Zappone et al., 2021).

### Sensitivity analysis

Sensitivity analyses were performed to assess the influence of the carbon footprint of the electricity mix, the CO_2_ transport mode, and the accounting of biogenic CO_2_ emissions on the overall carbon footprint of all scenarios.

#### Carbon footprint of the electricity mix

The abovementioned production processes require large amounts of electricity, and the corresponding carbon footprint can significantly influence the results (Kätelhön et al., 2019). Therefore, a sensitivity analysis for electricity was carried out. In contrast to the best-case scenario with offshore wind energy in the SSP2-RCP1.9 scenario, a sensitivity analysis was conducted in the SSP2-Base scenario for 2050 using high-carbon electricity from the German grid, assuming no additional climate policy in the future.

The electricity from the Swiss grid was adapted according to the business-as-usual scenario of the Swiss Energy Perspectives 2050+ (BFE, 2021). The carbon footprint of the electricity mixes used can be found in Table 2.

#### CO_2_ transport

A pipeline network for CO_2_ transportation has yet to be built, and construction poses many challenges, such as significant upfront investments, acceptance in local communities, and objections from landowners (Becattini et al., 2022; Energy Inc., 2014). Existing natural gas pipelines might be reused, but there are important differences in operating pressure and corrosion resistance when repurposing them for CO_2_ transport, which would require maintenance work (Onyebuchi et al., 2018). Alternative transportation methods have been suggested to bridge the gap between the availability of pipelines and current CCS projects (Becattini et al., 2022; Burger et al., 2024). To examine the impact of the CO_2_ transportation method on the overall results, an alternative transportation approach based on Burger et al. (2024) has been analyzed that involves a combination of trucks, inland barges, and ships to transport CO_2_ from Swiss WtE plants to a storage site in Northern Europe. Containers were used to transport the CO_2_, and the transportation distance was doubled since the containers needed to be returned to the capture site. A physical CO_2_ loss of 2.5% per 1000 km was assumed when using alternative transport modes (IPCC, 2005).

#### Accounting for biogenic CO_2_ emissions

In line with the conventional assumption, CO_2_ emissions are classified as climate neutral if the amount of CO_2_ emitted is balanced by the amount of CO_2_ sequestered from the atmosphere (IPCC, 2006). However, the accounting for biogenic CO_2_ emissions in climate change impacts is heavily discussed in the scientific literature, especially when the biomass is growing slowly (e.g., forest wood), due to the inherent time lag between harvest and regrowth (rotation period), as it takes time for the biomass to sequester the emitted CO_2_ (Cherubini et al., 2011; Guest et al., 2013; Levasseur et al., 2013). This leads to temporary increases in atmospheric CO_2_, which contributes to global warming and, therefore, would have a GWP between 0 and 1. This was considered in a sensitivity analysis.

Cherubini et al. (2011) calculated biogenic global warming potential (GWP_bio_) as a function of the biomass rotation period based on several simplifying assumptions, including that the harvest method is clear-cut, leading to a higher impact than other harvesting methods (Nabuurs et al., 2017). In the sensitivity analysis, we evaluate the variances in GWP_bio_ for different biomass feedstocks with rotation periods of 0–100 years. We take a conservative approach by neglecting the temporary storage of carbon in the anthroposphere by product use to assess the maximum potential impact of biogenic CO_2_ emissions on the overall carbon footprint.

S-6 contains additional sensitivities, such as the impact of the chosen future policy narrative, the capture technology in WtE plants, as well as the energy recovery efficiency in WtE plants.

## RESULTS

### Reference scenario

**FIGURE 2:**
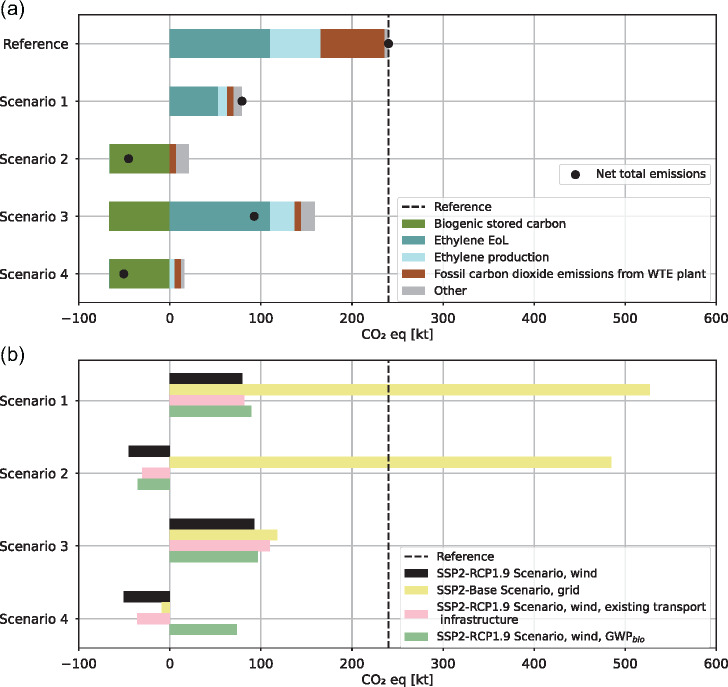
Carbon footprint of all scenarios. (a) Process contributions for all scenarios. The black dot represents net emissions. Assumptions: low-carbon wind electricity (carbon footprint: 9.434 kg CO_2_-eq/MWh), biogenic CO_2_ emission releases not considered (0 kg CO_2_-eq per kg), and biogenic CO_2_ storage benefit (−1 kg CO_2_ per kg biogenic CO_2_ stored permanently). (b) Sensitivity analysis. Variations caused by high-carbon electricity (yellow), alternative transport modes (red), and accounting for biogenic CO_2_ emissions (green) are presented in contrast to the results of the scenarios with wind energy and pipeline CO_2_ transport (black). The carbon footprint in the SSP2-RCP1.9 scenario is 9.434 CO_2_-eq/MWh, and the carbon footprint of the SSP2-Base scenario with grid electricity is 523.114 CO_2_-eq/MWh. The GWP_bio_ was taken from Cherubini et al. (2011) and has a factor of 0.43 (rotation period = 100 years). The carbon footprint in Scenario 4 can be somewhere between the black and green bars, depending on the biomass used. The data for all process contributions can be found in S-2. EoL, end-of-life; r, rotation period; WtE, waste-to-energy.

Without any carbon capture or emission reduction measures, the carbon footprint of the reference scenario has three main contributors: ethylene production, fossil ethylene EoL emissions, and fossil CO_2_ emissions from WtE plants (Figure 2a). The impact of the ethylene value chain is responsible for 70% of the carbon footprint in the reference scenario, more than double the impact of the WtE value chain.

### Climate mitigation strategies

**FIGURE 3:**
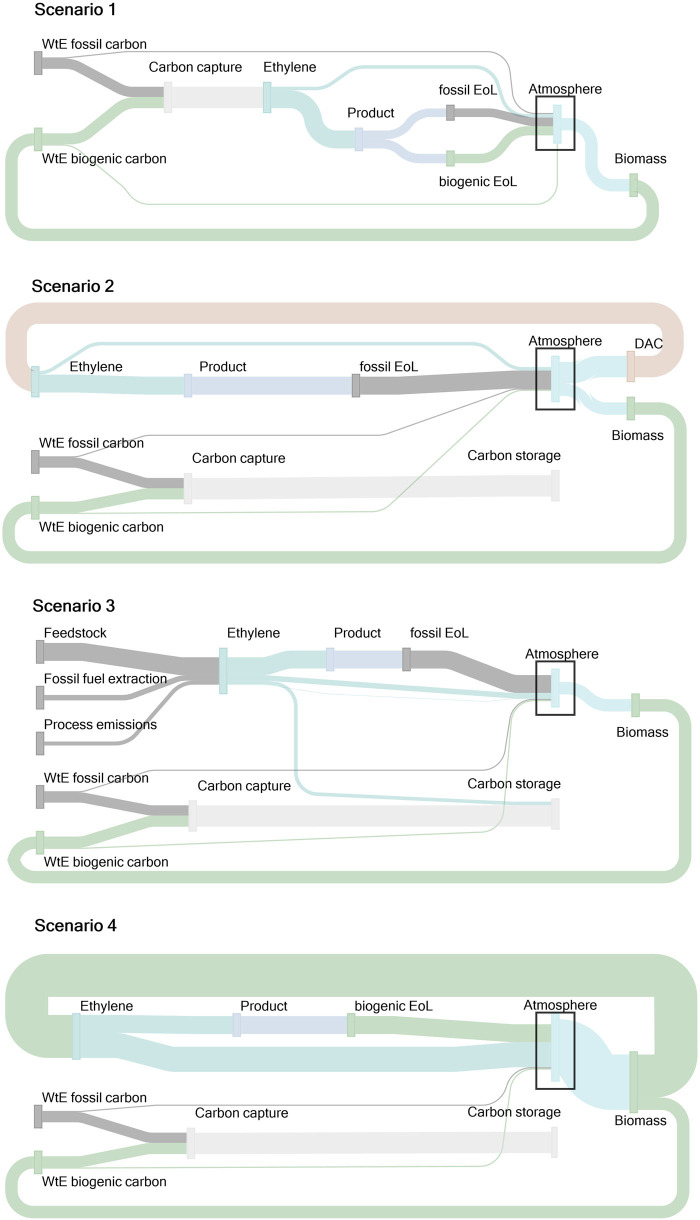
Biogenic and fossil carbon fluxes within the system. Biomass sequesters CO_2_ from the atmosphere. Biomass products that end up in waste-to-energy (WtE) plants produce biogenic CO_2_ emissions that are either emitted or captured. If more CO_2_ is sequestered from the atmosphere than emitted into the atmosphere, either through CO_2_ uptake of biomass or direct air capture (DAC), the overall carbon balance of the scenario is net negative. S-2 contains the data presented in this figure.

All scenarios reduce the carbon footprint of the reference scenario by at least 60%. Scenario 1 is the only instance where no biogenic CO_2_ emissions from the WtE plant are stored underground. Therefore, no negative emissions can be generated. In scenarios 2–4, the permanently stored biogenic CO_2_ compensates for emissions from production processes or residual emissions from WtE plants. In scenarios 2 and 4, EoL emissions are not associated with climate change impacts since the CO_2_ in the ethylene feedstock has been previously sequestered from the atmosphere, either by DAC or the regrowth of biomass (shown in Figure 3). This absence of fossil ethylene EoL emissions and low impacts from production allow for net negative emissions in those two scenarios.

The EoL emissions from ethylene with fossil feedstock have a significant share in the overall carbon footprint. When CO_2_ from WtE plants is utilized for ethylene production (scenario 1), 48% of the captured carbon dioxide counts as fossil emissions. The fossil ethylene EoL emissions could be reduced if the ratio of biogenic waste to fossil waste in the WtE plant increases or if the EoL emissions are captured again. The latter would occur if ethylene-derived materials like plastics are incinerated in a WtE plant with carbon capture.

Scenario 3 benefits primarily from the CCS in WtE plants. The capture and storage of biogenic carbon allows for negative emissions that can offset part of the positive emissions. Fossil emissions from WtE plants and steam cracking process emissions are reduced by 90% through CCS. However, the overall carbon footprint reduction in cradle-to-gate ethylene production is only about 42% compared to ethylene production without CCS. This is mainly attributed to the emissions related to fossil fuel extraction for the feedstock, which is still the same as in the reference scenario and is not altered through capturing the process emissions during steam cracking. The EoL emissions are not reduced if ethylene-derived products are combusted without CCS since the ethylene feedstock is fossil.

The flux of fossil and biogenic CO_2_ is presented in Figure 3. In the first two scenarios with ethylene from e-methanol, the CO_2_ needed per t of ethylene (3.75 t CO_2_ per t ethylene) exceeds the stoichiometric EoL emissions of ethylene (3.14 t CO_2_ per t ethylene). This is due to conversion inefficiencies during ethylene production, and the extra fossil emissions in scenario 1 were directly linked to the atmosphere and are included under “ethylene production” in Figure 2a. Similarly, in bio-methanol production, the CO_2_ from biomass for the production process surpasses the CO_2_ content in bio-methanol. The excess CO_2_ was emitted back into the atmosphere and classified as process emissions. Around 10% of the CO_2_ in WtE plants and the steam cracking process (in scenario 3) are not captured and released back into the atmosphere.

### Electricity consumption

**FIGURE 4:**
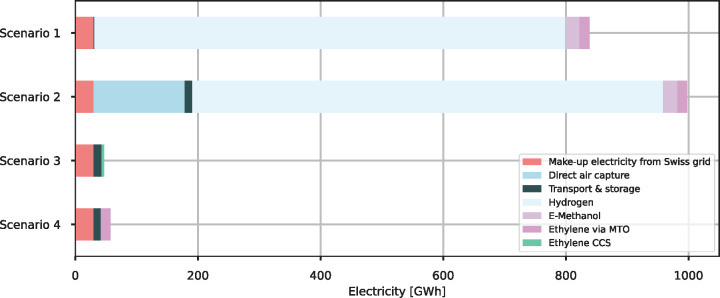
Electricity consumption for each scenario, in addition to the electricity consumption of ethylene production and waste-to-energy (WtE) operation in the reference scenario. The make-up electricity refers to the additional electricity that has to be fed into the Swiss grid to compensate for the electricity lost due to the carbon capture process in WtE plants. The data on electricity consumption of each scenario can be found in S-2. CCS, carbon capture and storage; MTO, methanol-to-olefin.

Electricity consumption shows a significant variation within the different scenarios (Figure 4). Decarbonization strategies using e-methanol (scenarios 1 and 2) consume the largest amount of electricity since the production of hydrogen with water electrolysis requires vast amounts of electricity. Ethylene production with DAC (scenario 2) has the highest electricity consumption due to the additional electricity needed in DAC, almost reaching 1000 GWh per FU (35,000t ethylene). Scenario 3 has the lowest electricity consumption of all scenarios, 21 times less than scenario 2 (47 GWh). Compared to the other processes, carbon capture in steam cracking and CO_2_ transport have a minor impact on electricity consumption.

The electricity consumption in DAC could be lowered by supplying waste heat to the capture process instead of producing heat with an HTHP, resulting in an electricity reduction of 68 GWh in Scenario 2.

### Sensitivity analysis

#### Electricity mix

When using high-carbon electricity from the German grid in ethylene production, the carbon footprint of scenarios 1 and 2 using the MTO production pathway with e-methanol increases substantially, in the first case more than doubling the carbon footprint of the reference scenario (Figure 2b). In the SSP2-Base scenario, 55% of the Western European electricity is generated by fossil fuels (Riahi et al., 2017; Sacchi et al., 2022; Stehfest et al., 2014). The difference from the low-carbon electricity scenario is significantly smaller in the other two scenarios with lower electricity requirements, especially in scenario 3, which has the lowest electricity consumption. Scenario 2 generates net negative emissions if the carbon footprint of the electricity mix falls below 56.3 kg CO_2_-eq/MWh.

#### Transportation method

Transporting CO_2_ with existing infrastructure has minor impacts on the overall carbon footprint (Figure 2b). Scenario 1 has the shortest transport distance since the captured CO_2_ from WtE plants is transported to the next petrochemical plant. The impact of the transport method is very similar in scenarios 2 and 4 and highest in scenario 3, where CO_2_ is captured in both industries and transported twice over long distances.

#### Accounting for biogenic CO_2_ emissions

Without accounting for the biogenic CO_2_ emissions, scenario 4 has a high climate mitigation potential of the assessed decarbonization trajectories. Depending on the rotation period of the biomass feedstock in ethylene production, scenario 4 reduces the carbon footprint of the reference scenario by 120%–69% (Figure 2b). The latter represents the maximum impact of biogenic CO_2_ emissions when biomass with long-rotation periods from clear-cut forests is used, and the CO_2_ is released immediately after harvest. Since biomass-based steam is used in the other scenarios (for the MTO process and CCS in steam cracking), there is a minor change in the overall carbon footprint due to the accounting of biogenic CO_2_ emissions. Biogenic CO_2_ emissions from WtE plants were excluded from the sensitivity analysis since most biogenic waste stems from short-rotation crops (e.g., food scraps or food waste), and the exact amount of long-rotation biomass (i.e., wood) in the waste and prior storage in the use phase (e.g., construction wood) is unknown (BAFU, 2022a). Depending on the share of long-rotation biomass, both the reference scenario and scenario 1 (CCU) could exhibit slightly changed biogenic GHG emissions.

## DISCUSSION

### Biogenic feedstock

Ethylene production with bio-methanol had a negative carbon footprint in the case of short-rotation biomass, but the carbon footprint increases and can become positive if long-rotation wood without temporary carbon storage is used as feedstock. Using biomass with a shorter rotation period seems intuitive since it can have a lower biogenic GWP due to the faster CO_2_ sequestration from the atmosphere. However, while in this paper, conservative assumptions were taken in the sensitivity analysis to assess biogenic CO_2_ emissions from existing forests and agriculture, land use change was not considered. Transforming old-grown forests to quick-rotation forests or even cropland can cause substantial permanent loss of carbon stock (Cooper, 1983; Dewar, 1991; Harmon et al., 1990; Schulze et al., 2000). On the other hand, Favero et al. (2023) argue that a higher biomass demand increases the price and leads to more afforestation and reforestation, positively affecting the carbon stored in forests. Therefore, it is unclear whether the additional biomass demand increases carbon emissions or creates additional carbon sinks through land use change (Havlík et al., 2011; Upham et al., 2009).

Besides climate change impacts, biobased chemicals may lead to burden shifts, particularly in terms of water scarcity and land use-related biodiversity loss. These impacts require careful assessment at the regional level to fully understand and mitigate their consequences (Huo et al., 2024).

### Renewable electricity demand

The second instance that can generate net negative emissions is scenario 2 with CO_2_ from DAC, but only if low-carbon electricity, such as offshore wind energy, is utilized. Scenario 2 is the pathway with the highest electricity consumption. Around 1 TWh of renewable electricity is required to produce the ethylene within the system boundaries of this analysis.

Various constraints, such as competition for land, public acceptance of energy infrastructure, and limitations of the electricity grid, limit the scale-up of renewable electricity and its infrastructure (Edenhofer et al., 2012). If renewable electricity is limited, other technologies with a higher CO_2_ reduction potential per kWh, like e-mobility and heat pumps, could be prioritized (Kätelhön et al., 2019).

### CCS in steam crackers

The results of the carbon footprint of scenario 3 with CCS in both industries are not substantially influenced by the parameters assessed in the sensitivity analysis, even if high-carbon electricity is used for ethylene production. The post-combustion carbon capture technology can be directly retrofitted to steam crackers, which might be particularly beneficial if the chemical production infrastructure has a few decades until the lifetime of the facility is reached. This allows for significant emissions reductions while using the existing infrastructure. However, CCS in steam crackers is not a long-term solution since the emissions from fossil fuel extraction remain, and the ethylene feedstock is still fossil, leading to a high impact of EoL emissions. Therefore, this solution cannot achieve net zero.

### CCU—Industrial symbiosis

Utilizing CO_2_ from WtE in ethylene production (scenario 1—CCU) displays industrial symbiosis, where CO_2_ as the by-product of WtE plants becomes a valuable resource for ethylene production. CCU has some benefits over CCS, namely fewer regulatory barriers, access to existing markets for chemicals, and lower costs due to shorter transport distances and the absence of storage costs (Brazzola et al., 2024; Eckle et al., 2021). Carbon removal certificates are the only products from CCS, and they are currently traded on voluntary carbon markets that are limited in size and dominated by cheaper negative emission technologies. In contrast, CCU products themselves are valuable and generate revenue (Brazzola et al., 2024; Höglund & Mitchell-Larson, 2022; Honegger, 2023; Styring et al., 2011). Additionally, public acceptance of CCU is higher than that of CCS (Arning et al., 2019; Dallo et al., 2024).

Scenario 1 is not a carbon-neutral system, mainly due to fossil EoL and process emissions. However, compared to the reference scenario, it reduces the carbon footprint by around two-thirds if low-carbon electricity is used. The CCU system described in this study might be an interim alternative until regulatory and economic barriers for CCS are overcome. In the long term, all chemical production processes should become carbon-neutral, and hence, the CO_2_ in the feedstock should originate from biomass or the air (IEA, 2023a).

### Costs

The expenses associated with carbon capture and low-carbon ethylene production pathways influence decision-making. Financial costs have been recognized as the primary obstacle in commercializing post-combustion carbon capture and subsequent carbon storage (Koytsoumpa et al., 2018; Leung et al., 2014). Poretti and Stengler (2022) highlighted the importance of a well-defined policy framework for the certification of negative emissions to make investing in CCS more attractive and expand its application.

Producing ethylene with the MTO process may only be feasible with low-cost methanol (IEA, 2018). E-methanol is responsible for 66%–81% of the total cost of ethylene, and its cost is highly dependent on green hydrogen, which is linked to the electricity price (Zhao et al., 2021). Current cost levels for e-methanol range from 720–2100 €/tonne, with CO_2_ from DAC on the higher end (990–2100 €/tonne) and CO_2_ from point sources (such as WtE plants) on the lower end (720–1400 €/tonne). The renewable methanol cost is expected to decrease with economies of scale and more mature technologies, but even at scale, ethylene from e-methanol could still surpass the European ethylene market price of 950–1250 €/tonne (IRENA, 2021; VCI, 2020). Additionally, current research suggests that future DAC costs could be about twice as much as currently estimated (Sievert et al., 2024). The cost of bio-methanol is linked to the price of biomass feedstock. Future biomass prices are expected to be higher than today due to increased demand, leading to increasing bio-ethylene prices (Havlik & Frank, 2023).

Table 3 summarizes the assessed decarbonization strategies, offering a comprehensive overview of the potential outcomes and implications.

**TABLE 3:** Advantages and obstacles of all scenarios.

Description	Ethylene feedstock	WtE	Advantages	Disadvantages
Scenario 1	CO_2_ (WtE)	CCU	• Shorter CO_2_ transport distances • Fewer regulatory barriers (CCU) • Lower cost than CCS or CO_2_ from DAC	• No negative CO_2_ emissions • High electricity consumption
Scenario 2	CO_2_ (DAC)	CCS	• Net negative emissions possible • DAC location flexible	• High electricity consumption • Potentially high costs
Scenario 3	Fossil	CCS	• Use of current infrastructure • Lower costs • Lower electricity consumption	• Emissions from feedstock extraction and EoL remain • Environmental impacts of fossil fuel extraction
Scenario 4	Biomass	CCS	• Net negative emissions possible • Lower electricity consumption	• Biomass availability at large scale • Potentially high biomass cost • Uncertain impacts of high biomass demand • Uncertain impact of biogenic CO_2_ emissions

Abbreviations: CCS, carbon capture and storage; CCU, carbon capture and utilization; DAC, direct air capture.

### Limitations

This study primarily assessed the carbon footprint of decarbonization strategies. Environmental impacts such as biodiversity loss, water consumption, ozone depletion, particulate matter emissions, and other decarbonization strategies like steam cracker electrification or different biomass-based ethylene production pathways were not evaluated. They could be included in further research projects. Additionally, a detailed economic assessment to compare the decarbonization strategies could provide essential insights for decision-makers.

The results provided in this paper are limited to the FU defined in Section 2.1. Resource constraints become essential when scaling up the assessed scenarios to the chemical production of a specific country or region, and a mix of the different decarbonization strategies is probably needed. Further research could investigate optimal resource allocations for carbon mitigation technologies and determine the optimal strategy mix for effective carbon reduction.

## CONCLUSION

This paper compared the carbon footprint of decarbonization strategies in the WtE and ethylene production. We demonstrate that carbon capture in WtE plants can lower the GHG emissions in the system, confirming the usefulness of the current policy. However, the overall system performance depends on downstream use or storage of the CO_2_ and other boundary conditions, such as the carbon intensity of the electricity mix. In particular, a low-carbon electricity mix is key for scenarios 1 and 2, in which CO_2_ from WtE carbon capture or DAC is used as feedstock for ethylene production. Due to the high electricity consumption of green hydrogen, producing ethylene via the MTO process is only climate-beneficial if low-carbon electricity is available; otherwise, emissions can even exceed the reference scenario. In such a scenario, the carbon captured in the WtE plant should rather be stored than utilized in the chemical industry. The same principle applies to DAC, as it is very energy-intensive and should primarily operate with low-carbon electricity.

Bio-ethylene production results in a negative carbon footprint, but sustainable biomass is a limited resource, and its demand is growing. Resource restrictions may make it necessary to include other decarbonization strategies on a larger scale. Additionally, the impact of biogenic EoL and process emissions is uncertain and difficult to assess, potentially increasing the overall climate change impact. Furthermore, biomass use comes with trade-offs in biodiversity loss, water stress, and competition in land use.

Steam cracking with CCS could be a viable option in the short-to-medium term since it allows steam crackers to be used until the end of their lifetime. However, it does not eliminate the impact of fossil fuel extraction or EoL incineration on climate change. Similarly, producing ethylene from captured carbon in WtE plants could be an interim strategy to lower emissions in both industries until regulatory and economic barriers to CCS deployment are overcome.

The inventory data of this study can easily be adapted to other regions, in case the basic technologies used are the same. The strategy implemented ultimately depends on site-specific constraints, available resources, and governing policies. Future research could focus on a detailed economic assessment of the different methods, include additional low-carbon ethylene production pathways, and assess the optimal mix of the decarbonization strategies on a regional or country level, considering particularly the energy mix and the availability of sustainable biomass resources.

## Supplementary Information

**Supporting Information S1**: This supporting information provides information on the calculations of carbon emissions in Waste-to-Energy plants, a detailed description of the future scenarios used, the life cycle inventories for the different technologies, and additional sensitivities and scenarios.

**Supporting Information S2**: This supporting information provides the model inputs for the different technologies and scenarios (exchanges) as well as data on the total carbon emissions and process contributions of the results that were used in Figures 2a and 2b in the manuscript. Additionally, the total electricity consumption (Figure 4) and fossil and biogenic carbon fluxes (Figure 3) within the system are presented.

## Data Availability

The data that supports the findings of this study are available in the supporting information of this article.
