# Supplementary material for: Assessing decarbonization strategies and industrial symbiosis in the chemical and waste-to-energy sector
Source: J Ind Ecol. 2025 Jan 23;29(2):486–502. doi: 10.1111/jiec.13616 (PMC13070073; doi:10.1111/jiec.13616)
Supplement: Supplementary file 1 — Supporting Information S1: This supporting information provides information on the calculations of carbon emissions in Waste-to-Energy plants, a detailed description of the future scenarios used, the life cycle inventories for the different technologies, and additional sensitivities and scenarios. [file 44498_2025_2902006_MOESM1_ESM.docx]

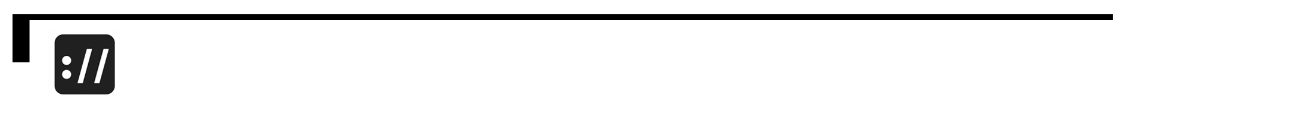


SUPPORTING INFORMATION FOR

Schnyder, M., Huo, J. & Hellweg, S. (2024): Assessing Decarbonization Strategies and Industrial Symbiosis in the Chemical and Waste-to-Energy Sector. *Journal of Industrial Ecology*.


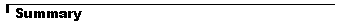


This supporting information provides information on calculating carbon emissions in Waste-to-Energy facilities, a detailed description of the future scenarios used, the life cycle inventories for the different technologies, and additional sensitivities and scenarios.


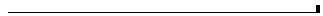


## S-1: CO_2_ emissions from Waste-to-Energy plants

Calculation of carbon content in waste (empirical correlation) (VBSA et al., 2021):

$C_{Total}\left[ \frac{g}{kg} \right]=264+\left( H_{u}\left[ \frac{MJ}{kg} \right]-10 \right)\times\frac{98}{5}$ (1)

$C_{Total}$: content of organic carbon (fossil and biogenic) in the waste

$H_{u}$: Lower heating value of waste, derived from steam production of boiler in Waste-to-Energy (WtE) plant

Fossil CO_2_ emissions:

${CO}_{2, fossil} \left[ \frac{g}{kg} \right]=\left( 0.48 \times C_{Total}\left[ \frac{g}{kg} \right] \right)\times\frac{44}{12}$ (2)

The fossil CO_2_ content in WtE emissions has been measured at the plant by determining the carbon isotope 14C, which is 48%. If the heating value is between 11 and 12 MJ per kg of waste, the fossil emission factor is between 499 and 534 g of fossil CO_2_ per kg of waste (VBSA et al., 2021).

## S-2: Scenario Description

The *ecoinvent* *3.9* database was transformed according to the SSP2-RCP1.9 Scenario through *premise* (ecoinvent, 2023; Sacchi et al., 2022; Wernet et al., 2016). Shared Socio-economic pathways (SSPs) describe alternative futures of developments in society, economy, and technology (O’Neill et al., 2014). SSP2 can be described as the middle-of-the-road scenario, and it follows development patterns from the past century (Fricko et al., 2017).

In the SSP2 scenario, social, economic, and technological trends are similar to today. Most economies are politically stable, with uneven development and income growth. Achieving the Sustainable Development Goals (SDGs) is slow, and environmental systems are degraded, but there are some improvements overall. Fossil fuels are still widely used, but dependency decreases slowly over time (Fricko et al., 2017; O’Neill et al., 2014). The global population will reach 9.4 billion people by 2070 and decline after that (KC & Lutz, 2017).

Within the SSP2 baseline, various levels of climate policy can be defined. The level of climate policy and subsequent climate mitigation can be characterized as the limit of anthropogenic radiative forcing (Representative Concentration Pathways, RCP) in 2100. This radiative forcing can span from 8.5 to 1.9W/m^2^, each translating to a specific amount of global warming (Fricko et al., 2017; Rogelj et al., 2018; Wayne, 2013). The IMAGE SSP2-Base scenario equals the RCP 6.0 scenario, where no additional climate policy is implemented in the future, leading to a Global Mean Surface Temperature (GMST) increase of 3.5 °C by 2100. In contrast, the SSP2-RCP1.9 scenario is a very optimistic scenario with a strong climate policy, resulting in a GMST increase of 1.2-1.4 °C by 2100 (Riahi et al., 2017; Rogelj et al., 2018; Sacchi et al., 2023). This scenario was chosen since those decarbonization transitions, especially green hydrogen production, are relevant in RCP1.9.

## S-3: Scenario Visualization

**Reference**

The Reference Scenario reflects current industry operations: neither industry captures carbon, and ethylene is produced through steam cracking. The End-of-Life (EoL) incineration emissions of final products from ethylene-derived products are fully accounted for.

**
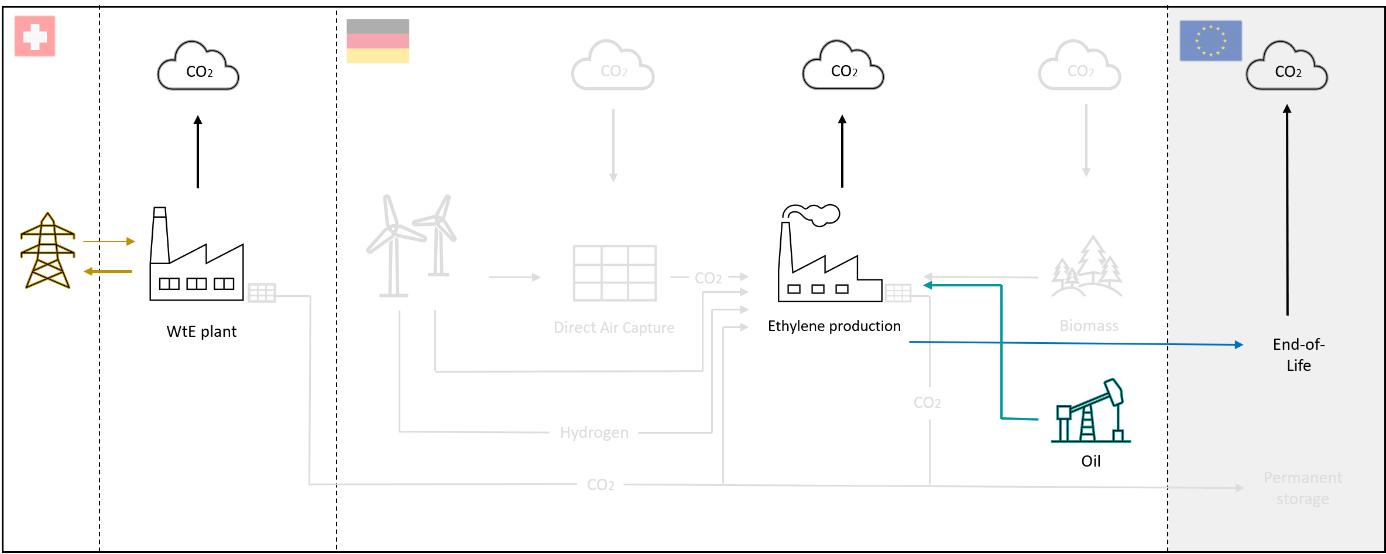
**

Figure S1: Reference Scenario.

**Scenario 1 – WtE with Carbon Capture and Utilization (CCU)**

CO_2_ captured from Swiss WtE plants and hydrogen produced from electrolysis are used as feedstock for CO_2_-based methanol (e-methanol) production, which is converted into ethylene via the Methanol-to-Olefin (MTO) process. Due to the energy penalty of carbon capture in WtE plants, additional electricity from the Swiss grid is required, with absorption heat pumps compensating for the lost heat. Ethylene with CO_2_ from WtE plants has reduced EoL emissions due to the share of biogenic carbon in the waste stream.

**
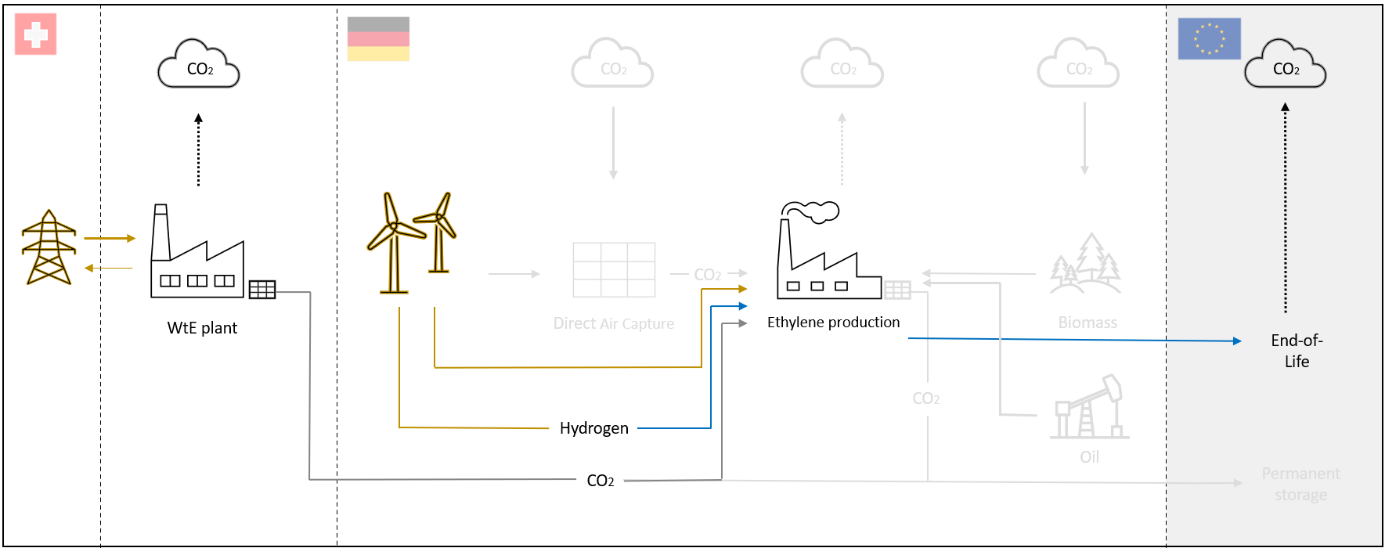
**

Figure S2: Scenario 1 - CCU in WtE.

**Scenario 2 – Ethylene with DAC**

CO_2_ is captured directly from the air with Direct Air Capture (DAC) and used together with green hydrogen to produce e-methanol and, eventually, ethylene via the MTO process. Since the CO_2_ was initially taken from the atmosphere, no EoL emissions are considered. All emissions from WtE plants are captured and stored in Northern Europe. Lost electricity during the capture process is compensated for by electricity from the Swiss grid, and heat pumps provide additional heat.

**
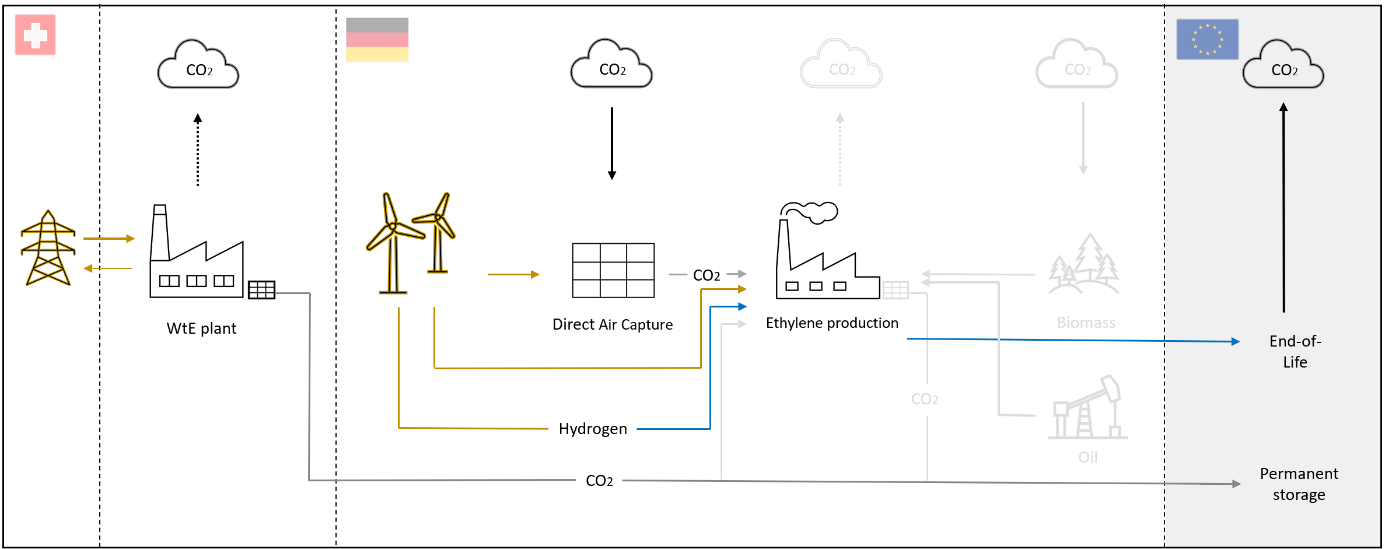
**

Figure S3: Scenario 2 - DAC-based ethylene.

**Scenario 3 – Steam cracking with CCS**

Ethylene is produced via steam cracking of naphtha, and the process emissions are captured, transported to Northern Europe, and stored underground. CO_2_ emissions from WtE plants are also captured and stored. EoL emissions are fully accounted for.

**
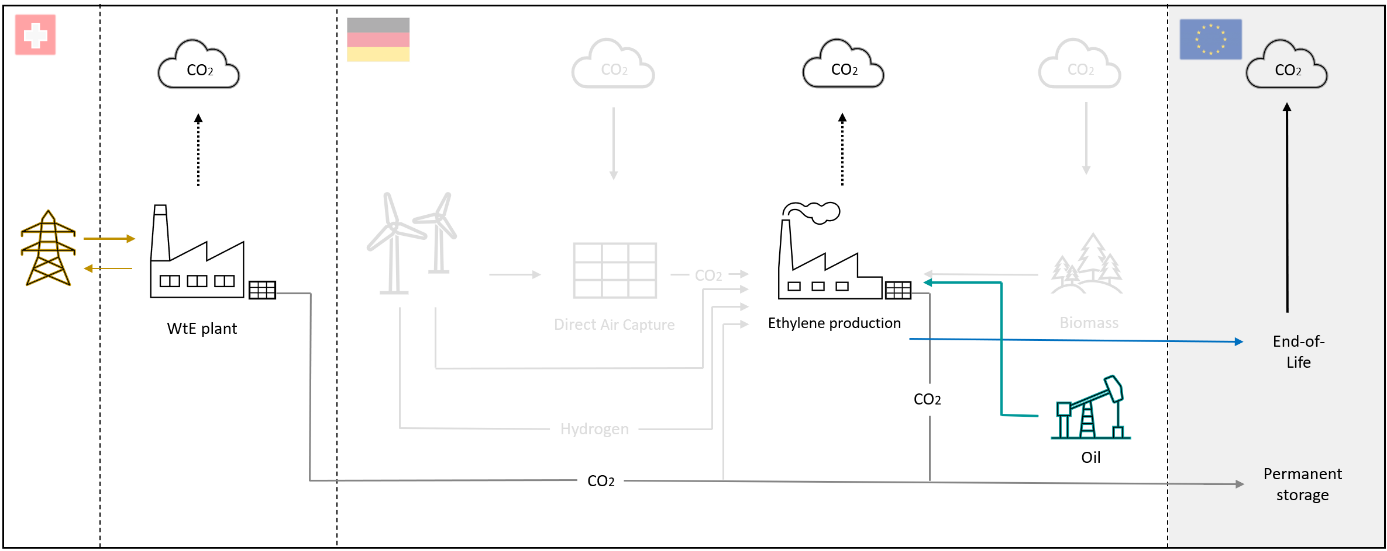
**

Figure S4: Scenario 3 - Steam cracking with CCS.

**Scenario 4 – Bio-Ethylene**

Lignocellulosic biomass is gasified to produce bio-methanol, which is converted into ethylene via the MTO process. Emissions from WtE plants are captured and permanently stored. No EoL emissions are accounted for since the feedstock is biogenic.

**
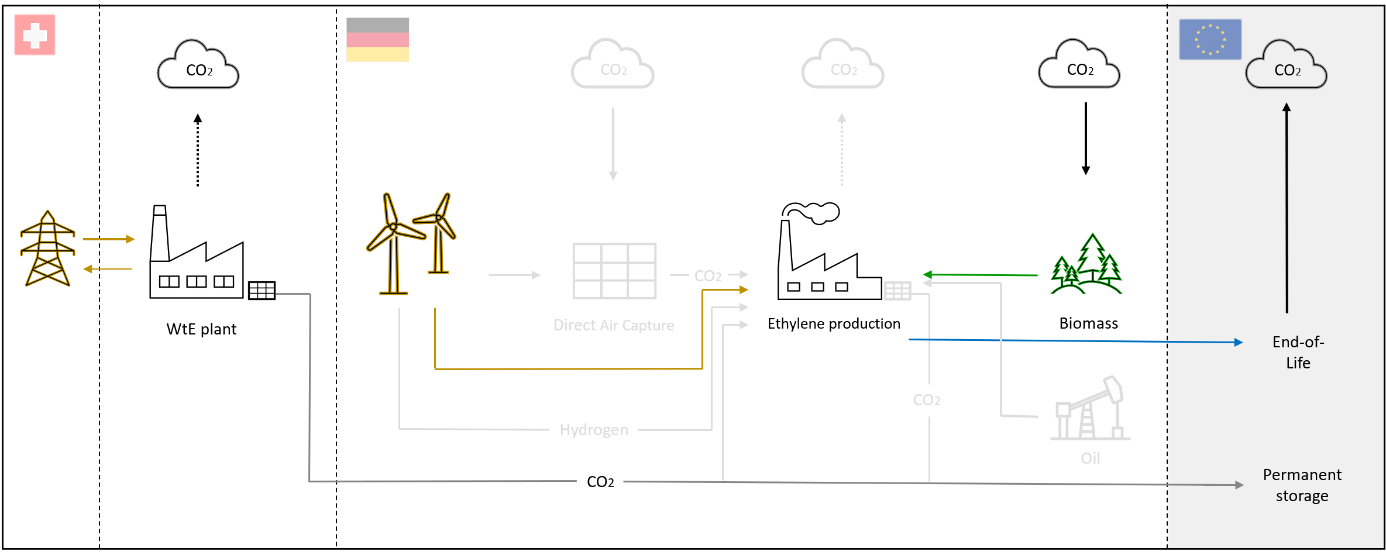
**

Figure S5: Scenario 4 – Bio-ethylene.

## S-4: Life Cycle Inventories

Table S1: Life Cycle Inventory (LCI) of ethylene production processes

| **Product** | **Input** | **Process** | **Process emissions** | **Source** |
| --- | --- | --- | --- | --- |
| 1 kg hydrogen | 44 kWh electricity (future)  50 kWh electricity (current)  9 kg water | Water electrolysis |  | Bauer et al. (2022)  Hoppe et al. (2018)  Sacchi et al. (2023) |
| 1 kg ethylene | 1.43 kg naphtha  0.38 Wh electricity  56.8 g natural gas  4.8 g sodium hydroxide | Steam cracking with naphtha | 0.70 kg CO_2_ | Keller et al. (2020) |
| 1 MJ steam | 0.064 kg biomass^1^ | Biomass boiler | 0.32 kg CO_2_^2^ | Pérez-Uresti et al. (2019) |
| 1 ton kilometer pipeline transport with recompression | 0.038 kWh electricity | Supercritical CO_2_ pipeline transport with recompression |  | Terlouw et al. (2021) |
| 1 kg CO_2_ from DAC | 1.13 kWh electricity  (0.61 kWh electricity,  5.4 MJ waste heat)^3^ | Solid sorbent low-temperature DAC with HTHP (waste heat) + CO_2_ compression |  | Terlouw et al. (2021) |
| 1 kg CO_2_ captured from WtE  1 kg CO_2_ captured from steam cracker | 3.348 MJ steam  0.050 kWh electricity^4^  0.114 kWh electricity^5^  0.086 kWh electricity^6^  0.024 kWh electricity^7^  3.695 MJ steam  0.073 kWh electricity^4^  0.114 kWh electricity^5^  0.086 kWh electricity^6^  0.024 kWh electricity^7^ | Post-combustion MEA capture in WtE plants  Post-combustion MEA capture in steam cracker |  | Industrial Data  Ros et al. (2022)  Suviranta (2023)  Terlouw et al. (2021)  Hu et al. (2023)  Suviranta (2023)  Terlouw et al. (2021) |
| 1 kg e-methanol | 0.193 kg hydrogen  1.457 kg CO_2_  0.258 kWh electricity | Methanol synthesis via CO_2_ hydrogenation |  | González-Garay et al. (2019)  Khojasteh-Salkuyeh et al. (2021)  Nizami et al. (2022) |
| 1 kg bio-methanol | 1.595 kg dry biomass^1^  2.95 kg water  0.054 MJ steam^8^ | Methanol synthesis via biomass gasification | 1.71 kg CO_2_^2^ | de Fournas and Wei (2022) |
| 1 kg ethylene | 2.571 kg methanol  0.458 kWh electricity  5.59 MJ steam | MTO Process |  | Hoppe et al. (2018)  Xiang et al. (2014) |

^1^ cleft timber, measured as dry mass; softwood forestry, spruce, sustainable forest management (ecoinvent 3.9)

^2^ Biogenic CO_2_

^3^ DAC with heat from waste heat

^4^ electricity for capture unit

^5^ electricity for compression (pipeline transport, from 1 to 80 bar)

^6^ electricity for liquefaction, at -22 °C, to -28 °C, 15-18 bars

^7^ electricity for CO_2_ storage

^8^ assumption 1 kg steam = 2.75 MJ (ecoinvent, 2023)

## S-5: Additional Scenarios

Two additional scenarios were analyzed to assess the impact of only one of the two industries reducing its emissions (WtE in Scenario 5 and the chemical industry in Scenario 6).

**Scenario 5**

Only the WtE industry reduces its emissions by capturing and storing CO_2_. Ethylene is still created through steam cracking without carbon capture. Due to the energy penalty of carbon capture, additional electricity from the Swiss grid is required. Absorption heat pumps compensate for heat lost in the capture process, and EoL emissions are fully accounted for.

**Scenario 6**

Only the chemical industry decarbonizes, producing ethylene with CO_2_ from DAC, while the WtE industry does not capture or store carbon emissions. EoL emissions are not considered since the CO_2_ was initially taken from the atmosphere.

If low-carbon electricity is used, Scenario 6, with the decarbonization of ethylene production, has a lower carbon footprint than Scenario 5, where only the WtE plant reduces its emissions. The results in Scenario 5 do not change substantially with the assessed sensitivities, whereas the carbon footprint of Scenario 6 is affected by the electricity mix (Figure S6).


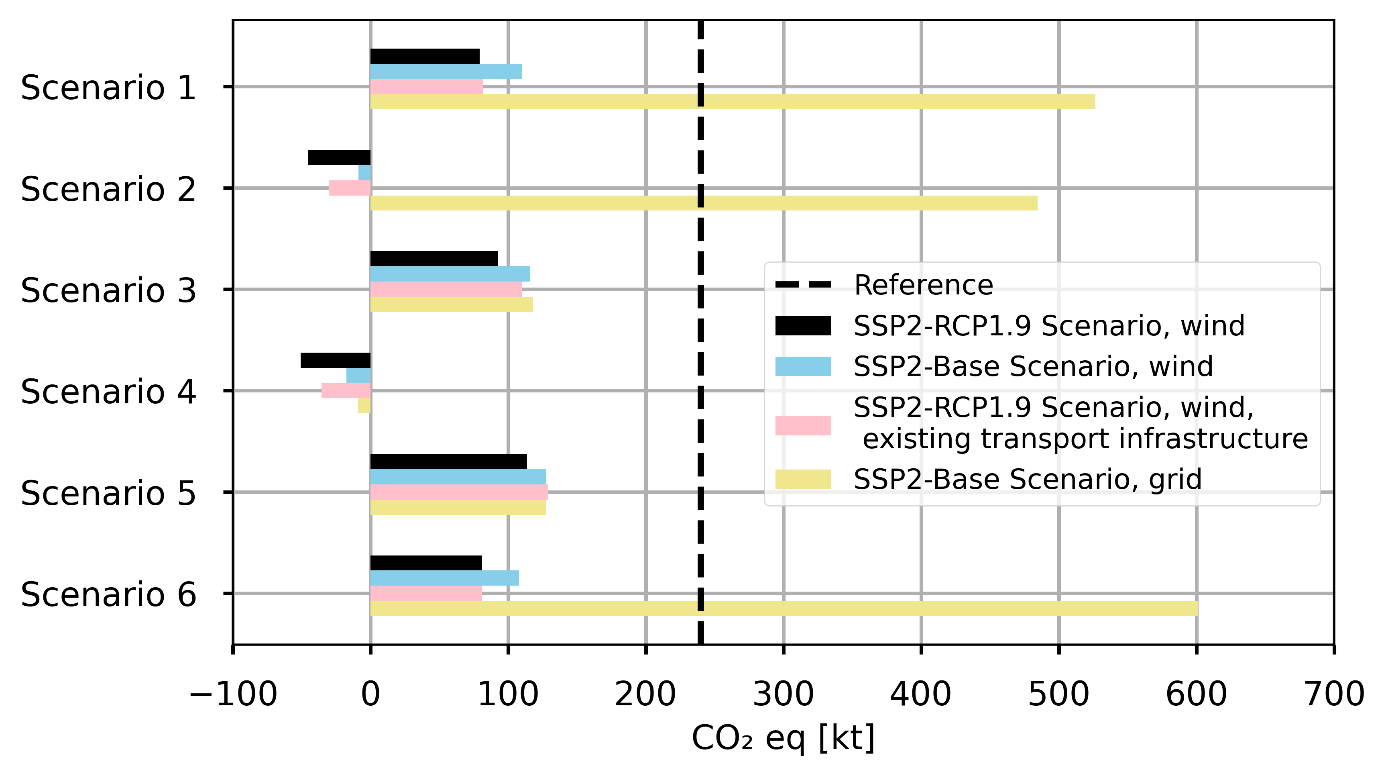


Figure S6: GWP for 100 years, including sensitivities for all six scenarios. Wind electricity in the SSP2-RCP1.9 scenario has a carbon footprint of 9.434kg CO2-eq per MWh, and in the SSP2-Base scenario, 14.746 kg CO_2_-eq per MWh. The carbon footprint of grid electricity in the SSP2-Base scenario is 523.114 kg CO_2_-eq per MWh.

The carbon fluxes of the two additional cases are shown in Figure S7 below. The carbon flux of Scenario 5 is very similar to Scenario 3, and the only difference is the captured process emissions during steam cracking.


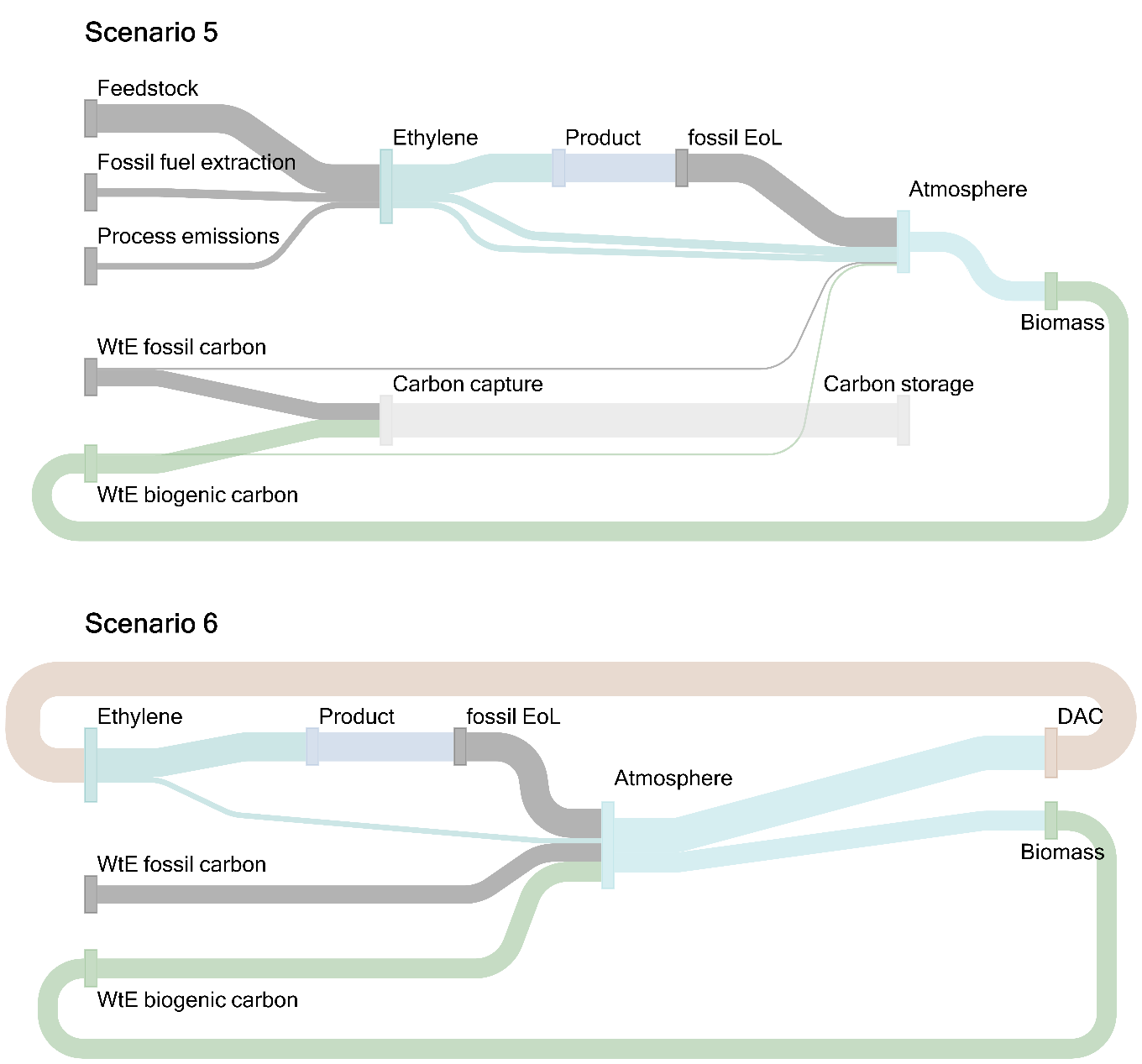


Figure S7: Biogenic and fossil carbon fluxes for Scenarios 5 and 6. Biomass sequesters CO_2_ from the atmosphere. Biomass products that end up in WtE plants produce biogenic emissions that are either emitted or captured. If more CO_2_ is sequestered from the atmosphere than emitted into the atmosphere, either through CO_2_ uptake of biomass or DAC, the overall carbon balance of the case is net negative.

## S-6: Additional Sensitivities

**Policy Scenarios**

The background database has been transformed through premise according to the IMAGE SSP2-RCP1.9 scenario. Since this scenario depicts the best possible outcome, the LCA was also conducted using the IMAGE SSP2-Base scenario, which assumes overall stabilizing emissions without additional climate policy, leading to a global temperature increase of 3.5 °C (Riahi et al., 2017; Sacchi et al., 2023). The aim is to assess the effect of the background dataset on the results.


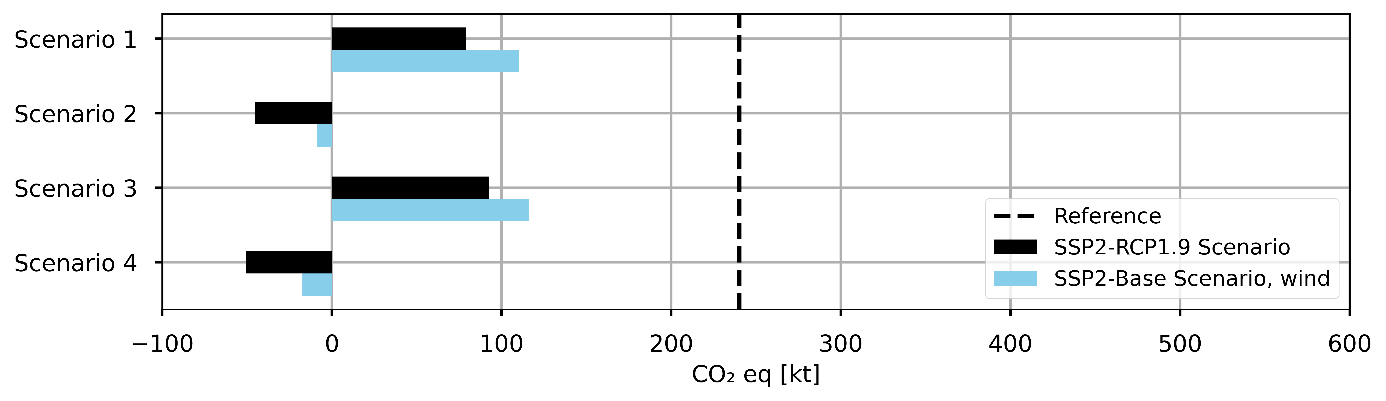


Figure S8: Effect of change in background scenario on the results. Wind electricity in the SSP2-RCP1.9 scenario has a carbon footprint of 9.434 kg CO_2_-eq per MWh, and in the SSP2-Base scenario, 14.746 kg CO_2_-eq per MWh.

As illustrated in Figure S8, the choice of the background system affects the total carbon footprint of the scenarios. This can be attributed to efforts to decarbonize the underlying processes, such as steel production or transitioning to a low-carbon electricity mix that affects the carbon footprint of the background data. The increase in carbon footprint is consistent across all scenarios and does not change the order of the scenarios.

**Capture Technology**

Amine-based post-combustion carbon capture technologies are currently the most used capture technology for industrial point sources in Europe (Poretti & Stengler, 2022). Monoethanolamine (MEA) has been found to have the highest CO_2_-carrying capacity by mass among amines (Zhao et al., 2013). However, the degradation of the solvent could lead to potential health hazards, and its impact needs to be considered (Grant et al., 2014). Other solvents for post-combustion carbon capture, like Hot Potassium Carbonate (HPC), are emerging and could be alternatives to MEA (Shear, 2023).

Both the MEA and HPC capture technology are considered for capturing carbon at the future CCS pilot plant in Swiss WtE plants. While MEA carbon capture requires electricity and steam to operate, the HPC technology solely needs electricity for capture and can produce additional heat for the district heating system (Shear, 2023). This extra heat delivered to the district heating system was accounted for as an avoided burden.

HPC as a capture method in Swiss WtE plants did not change the overall carbon footprint (Figure S9), even though the technology requires more electricity than MEA to capture the same amount of carbon. This is because the electricity for capture originates directly from the WtE plant, and the additional electricity from the Swiss grid fed into the system to compensate for the electricity penalty has a comparatively low carbon footprint. The additional district heat fed into the system and counted as an avoided burden did not change the results at scale.

The results differ in countries with a high-carbon electricity mix. Figure S10 presents the carbon footprint if the WtE plant was located in Germany, and the make-up electricity originated from the German electricity grid in the SSP2-Base scenario. The electricity for the ethylene production chain was still wind energy to isolate electricity impact on the WtE system.

Generating more heat and steam in the HPC capture process could lead to higher electricity production within the plant, meaning that less electricity from the grid is required to compensate for electricity losses. The overall change in carbon footprint due to the capture technology is minor compared to the impact of the electricity mix.


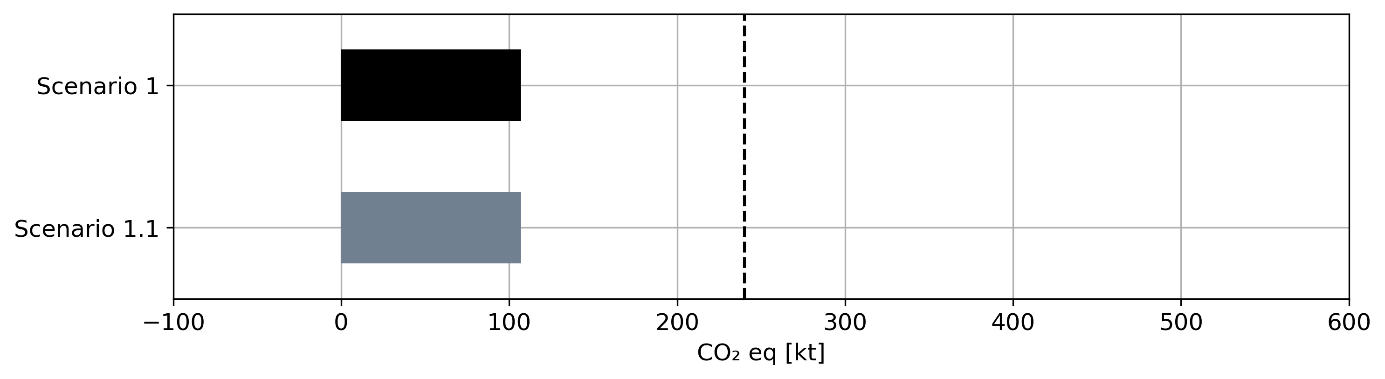


Figure S9: GWP 100 in SSP2-RCP1.9 Scenario. Scenario 1: MEA carbon capture technology. Scenario 1.1: HPC carbon capture technology. WtE plant is located in Switzerland.


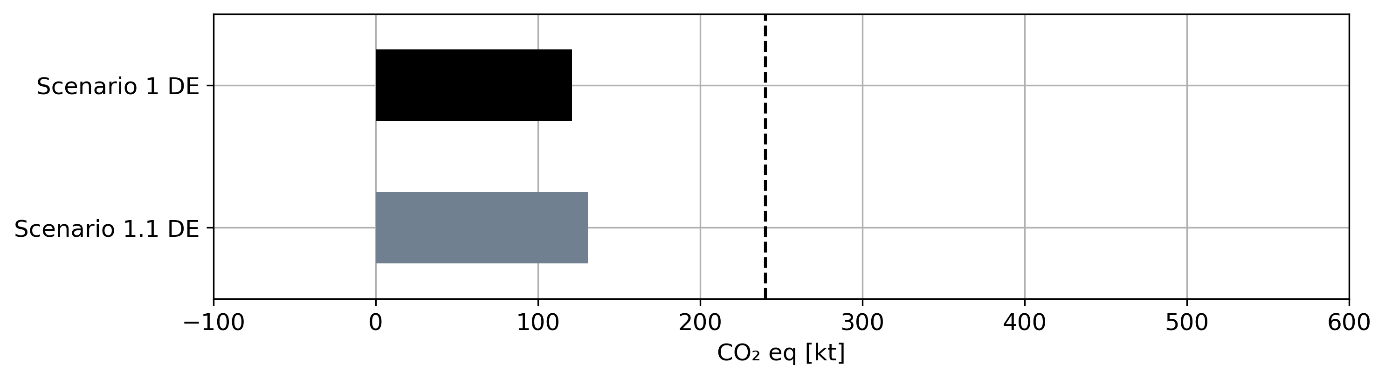


Figure S10: GWP 100 in SSP2-Base Scenario. Scenario 1: MEA carbon capture technology. Scenario 1.1: HPC carbon capture technology. WtE plant is located in Germany. Only the make up electricity in WtE plants is from the German grid, the electricity for ethylene is wind energy.

**High energy recovery rates in WTE plant**

The previous calculations are based on the energy recovery of an average Swiss WtE plant. Many Swiss WtE plants have been operating for decades and do not have optimal energy recovery. However, we conducted a sensitivity analysis assuming higher efficiency levels of future Swiss WtE plants, recovering more heat and electricity from the same amount of waste. New WtE plants have a gross thermal efficiency of as high as 98% of the lower heating value (LHV) (around 25% of electricity production and 73% of heat recovery, compared to the current average of 18% of electricity production and 32% heat recovery) (Bisinella et al., 2022). The additional electricity and heat from the increased efficiency of the WtE plants were accounted for as avoided burdens.


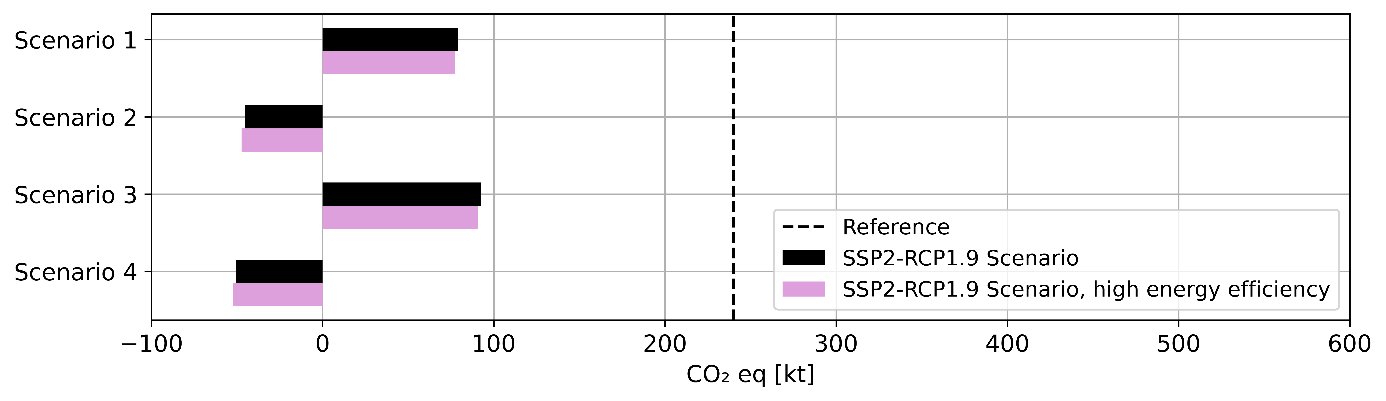


Figure S11: Carbon footprint comparison of the scenarios with the efficiency of an average Swiss WtE plant (black) and scenarios with a high energy efficiency in WtE plants (pink) in the SSP2-RCP1.9 Scenario.

Figure S11 shows a small decrease of the carbon footprint in all scenarios, which is due to the avoided burden of the additional electricity and district heat produced through the increased energy efficiency. This difference is more visible in the scenario with a high-carbon electricity mix (Figure S12).


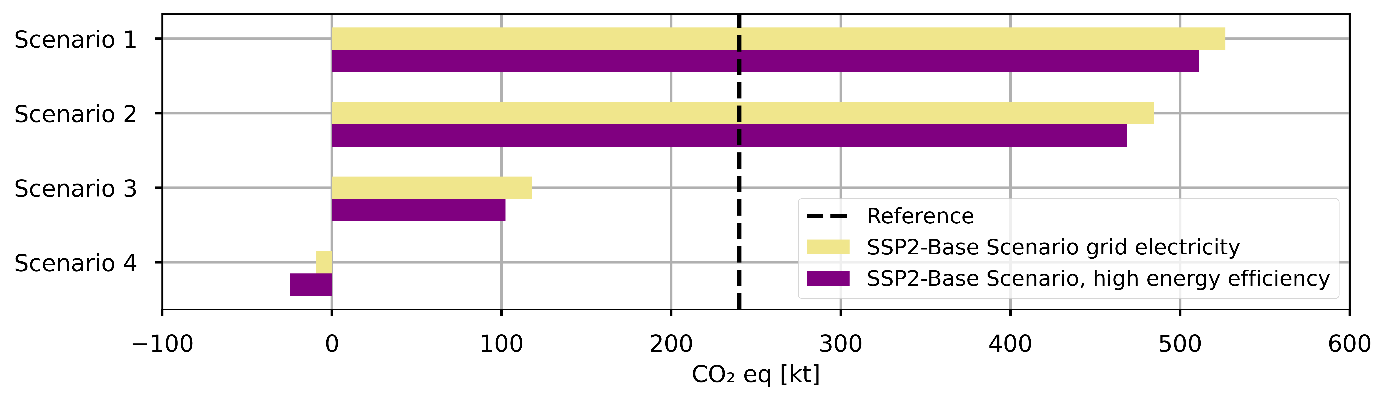


Figure S12: Carbon footprint comparison of the scenarios with the efficiency of an average Swiss WtE plant (yellow) and scenarios with high energy efficiency in WtE plants (purple) in the SSP2-Base Scenario.

According to industry experts, the current electricity penalty of carbon capture in WtE plants could be as much as 60-80%, meaning that through carbon capture, the WtE plant could supply 60-80% less electricity to the Swiss grid, regardless of capture technology. While this might be an important aspect for the WtE business and its economics, there are only minor impacts on the carbon footprint since Switzerland has a low-carbon electricity mix.

## References

Bauer, Christian, Harshil Desai, Thomas Heck, Romain Sacchi, Simon Schneider, Tom Terlouw, Karin Treyer, and Xiaojin Zhang. 2022. *Stromspeicherung Und Wasserstoff - Technologien, Kosten Und Auswirkungen Auf Das Klima*.

Bisinella, V., Nedenskow, J., Riber, C., Hulgaard, T., Christensen, T. 2022. “Environmental Assessment of amending the Amager Bakke incineration plant in Copenhagen with carbon capture and storage.” *Waste Management and Research* 40(1), 79-95. doi: 10.1177/0734242X211048125

ecoinvent. 2023. “Ecoinvent Version 3.9.” Retrieved May 30, 2024 (https://support.ecoinvent.org/ecoinvent-version-3.9).

de Fournas, Nicolas, and Max Wei. 2022. “Techno-Economic Assessment of Renewable Methanol from Biomass Gasification and PEM Electrolysis for Decarbonization of the Maritime Sector in California.” *Energy Conversion and Management* 257:115440. doi: 10.1016/j.enconman.2022.115440.

Fricko, Oliver, Petr Havlik, Joeri Rogelj, Zbigniew Klimont, Mykola Gusti, Nils Johnson, Peter Kolp, Manfred Strubegger, Hugo Valin, Markus Amann, Tatiana Ermolieva, Nicklas Forsell, Mario Herrero, Chris Heyes, Georg Kindermann, Volker Krey, David McCollum, Michael Obersteiner, Shonali Pachauri, Shilpa Rao, Erwin Schmid, Wolfgang Schoepp, and Keywan Riahi. 2017. “The Marker Quantification of the Shared Socioeconomic Pathway 2: A Middle-of-the-Road Scenario for the 21st Century.” *Global Environmental Change* 42:251–67. doi: 10.1016/j.gloenvcha.2016.06.004.

González-Garay, Andrés, Matthias S. Frei, Amjad Al-Qahtani, Cecilia Mondelli, Gonzalo Guillén-Gosálbez, and Javier Pérez-Ramírez. 2019. “Plant-to-Planet Analysis of CO2-Based Methanol Processes.” *Energy and Environmental Science* 12(12):3425–36. doi: 10.1039/c9ee01673b.

Grant, Tim, Clare Anderson, and Barry Hooper. 2014. “Comparative Life Cycle Assessment of Potassium Carbonate and Monoethanolamine Solvents for CO2 Capture from Post Combustion Flue Gases.” *International Journal of Greenhouse Gas Control* 28:35–44. doi: 10.1016/j.ijggc.2014.06.020.

Hoppe, Wieland, Nils Thonemann, and Stefan Bringezu. 2018. “Life Cycle Assessment of Carbon Dioxide–Based Production of Methane and Methanol and Derived Polymers.” *Journal of Industrial Ecology* 22(2):327–40. doi: 10.1111/jiec.12583.

Hu, Guihua, Xiaoxu Li, Xiaoyan Liu, Jun Hu, Olajide Otitoju, Meihong Wang, Wenli Du, Zhencheng Ye, Jian Long, and Feng Qian. 2023. “Techno-Economic Evaluation of Post-Combustion Carbon Capture Based on Chemical Absorption for the Thermal Cracking Furnace in Ethylene Manufacturing.” *Fuel* 331:125604. doi: 10.1016/J.FUEL.2022.125604.

KC, Samir, and Wolfgang Lutz. 2017. “The Human Core of the Shared Socioeconomic Pathways: Population Scenarios by Age, Sex and Level of Education for All Countries to 2100.” *Global Environmental Change* 42:181–92. doi: 10.1016/J.GLOENVCHA.2014.06.004.

Keller, Florian, Roh Pin Lee, and Bernd Meyer. 2020. “Life Cycle Assessment of Global Warming Potential, Resource Depletion and Acidification Potential of Fossil, Renewable and Secondary Feedstock for Olefin Production in Germany.” *Journal of Cleaner Production* 250:119484. doi: 10.1016/J.JCLEPRO.2019.119484.

Khojasteh-Salkuyeh, Yaser, Omid Ashrafi, Ehsan Mostafavi, and Philippe Navarri. 2021. “CO2utilization for Methanol Production; Part I: Process Design and Life Cycle GHG Assessment of Different Pathways.” *Journal of CO2 Utilization* 50:101608. doi: 10.1016/j.jcou.2021.101608.

Nizami, Muhammad, Slamet, and Widodo Wahyu Purwanto. 2022. “Solar PV Based Power-To-Methanol via Direct CO2hydrogenation and H2O Electrolysis: Techno-Economic and Environmental Assessment.” *Journal of CO2 Utilization* 65:102253. doi: 10.1016/j.jcou.2022.102253.

O’Neill, Brian C., Elmar Kriegler, Keywan Riahi, Kristie L. Ebi, Stephane Hallegatte, Timothy R. Carter, Ritu Mathur, and Detlef P. van Vuuren. 2014. “A New Scenario Framework for Climate Change Research: The Concept of Shared Socioeconomic Pathways.” *Climatic Change* 122(3):387–400. doi: 10.1007/S10584-013-0905-2/TABLES/2.

Pérez-Uresti, Salvador I., Mariano Martín, and Arturo Jiménez-Gutiérrez. 2019. “Estimation of Renewable-Based Steam Costs.” *Applied Energy* 250:1120–31. doi: 10.1016/j.apenergy.2019.04.189.

Poretti, Fabio, and Ella Stengler. 2022. “The Climate Roadmap of the European Waste-to-Energy Sector | The Path to Carbon Negative.” *SSRN Electronic Journal*. doi: 10.2139/SSRN.4284664.

Riahi, Keywan, Detlef P. van Vuuren, Elmar Kriegler, Jae Edmonds, Brian C. O’Neill, Shinichiro Fujimori, Nico Bauer, Katherine Calvin, Rob Dellink, Oliver Fricko, Wolfgang Lutz, Alexander Popp, Jesus Crespo Cuaresma, Samir KC, Marian Leimbach, Leiwen Jiang, Tom Kram, Shilpa Rao, Johannes Emmerling, Kristie Ebi, Tomoko Hasegawa, Petr Havlik, Florian Humpenöder, Lara Aleluia Da Silva, Steve Smith, Elke Stehfest, Valentina Bosetti, Jiyong Eom, David Gernaat, Toshihiko Masui, Joeri Rogelj, Jessica Strefler, Laurent Drouet, Volker Krey, Gunnar Luderer, Mathijs Harmsen, Kiyoshi Takahashi, Lavinia Baumstark, Jonathan C. Doelman, Mikiko Kainuma, Zbigniew Klimont, Giacomo Marangoni, Hermann Lotze-Campen, Michael Obersteiner, Andrzej Tabeau, and Massimo Tavoni. 2017. “The Shared Socioeconomic Pathways and Their Energy, Land Use, and Greenhouse Gas Emissions Implications: An Overview.” *Global Environmental Change* 42:153–68. doi: 10.1016/J.GLOENVCHA.2016.05.009.

Rogelj, Joeri, Alexander Popp, Katherine V. Calvin, Gunnar Luderer, Johannes Emmerling, David Gernaat, Shinichiro Fujimori, Jessica Strefler, Tomoko Hasegawa, Giacomo Marangoni, Volker Krey, Elmar Kriegler, Keywan Riahi, Detlef P. Van Vuuren, Jonathan Doelman, Laurent Drouet, Jae Edmonds, Oliver Fricko, Mathijs Harmsen, Petr Havlík, Florian Humpenöder, Elke Stehfest, and Massimo Tavoni. 2018. “Scenarios towards Limiting Global Mean Temperature Increase below 1.5 °C.” *Nature Climate Change 2018 8:4* 8(4):325–32. doi: 10.1038/s41558-018-0091-3.

Ros, Jasper, Roberta Veronezi Figueiredo, Tanya Srivastava, Arjen Huizinga, Peter van Os, Hans Wassenaar, and Juliana Garcia Moretz-Sohn Monteiro. 2022. “Results of the 2020 and 2021 Campaigns of the Commercial Carbon Capture Plant at AVR Duiven.” *SSRN Electronic Journal*. doi: 10.2139/ssrn.4282665.

Sacchi, R., T. Terlouw, K. Siala, A. Dirnaichner, C. Bauer, B. Cox, C. Mutel, V. Daioglou, and G. Luderer. 2022. “PRospective EnvironMental Impact AsSEment (Premise): A Streamlined Approach to Producing Databases for Prospective Life Cycle Assessment Using Integrated Assessment Models.” *Renewable and Sustainable Energy Reviews* 160:112311. doi: 10.1016/j.rser.2022.112311.

Sacchi, Romain, Alois Dirnaichner, Chris Mutel, and Brian Cox. 2023. “In a Nutshell — Premise 1.7.2 Documentation.” Retrieved January 17, 2024 (https://premise.readthedocs.io/en/latest/introduction.html).

Shear, M. Katherine. 2023. *State of the Art : CCS Technologies 2022*. Vol. 14.

Suviranta, Roosa. 2023. “Carbon Capture Integration to Steam Cracker Furnaces - Techno-Economic Evaluation.” LUT School of Engineering Science.

Terlouw, Tom, Karin Treyer, Christian Bauer, and Marco Mazzotti. 2021. “Life Cycle Assessment of Direct Air Carbon Capture and Storage with Low-Carbon Energy Sources.” *Environmental Science and Technology* 55(16):11397–411. doi: 10.1021/acs.est.1c03263.

VBSA, BAFU, and BFE. 2021. *Monitoring-Bericht Zur CO 2-Branchenvereinbarung Für Das Jahr 2020*. Bern.

Wayne, Graham. 2013. *The Beginner’s Guide to Representative Concentration Pathways*.

Wernet, Gregor, Christian Bauer, Bernhard Steubing, Jürgen Reinhard, Emilia Moreno-Ruiz, and Bo Weidema. 2016. “The Ecoinvent Database Version 3 (Part I): Overview and Methodology.” *International Journal of Life Cycle Assessment* 21(9):1218–30. doi: 10.1007/S11367-016-1087-8/METRICS.

Xiang, Dong, Siyu Yang, Xia Liu, Zihao Mai, and Yu Qian. 2014. “Techno-Economic Performance of the Coal-to-Olefins Process with CCS.” *Chemical Engineering Journal* 240:45–54. doi: 10.1016/j.cej.2013.11.051.

Zhao, Ming, Andrew I. Minett, and Andrew T. Harris. 2013. “A Review of Techno-Economic Models for the Retrofitting of Conventional Pulverised-Coal Power Plants for Post-Combustion Capture (PCC) of CO2.” *Energy and Environmental Science* 6(1):25–40. doi: 10.1039/c2ee22890d.
